# Air purifiers, comparison between real and declared surface for use: fake it or make it?

**DOI:** 10.1093/eurpub/ckac131.249

**Published:** 2022-10-25

**Authors:** C Gullotto, L Dragoni, D Amodeo, G Cevenini, N Nante, G Messina

**Affiliations:** 1 Hygiene and Preventive Medicine, University of Siena, Siena, Italy; 2 Department of Medical Biotechnologies, University of Siena, Siena, Italy; 3 Department of Molecular and Developmental Medicine, University of Siena, Siena, Italy

## Abstract

**Introduction:**

Air pollution has been recognized as one of the major risk factors for the global burden of disease. In modern society, most exposure occurs indoors, and air quality may be improved with air purifiers utilizing various cleaning techniques. This analysis aims to evaluate whether recommended room surface in which to use these devices as declared by producers is actually in line with their real effective area of activity.

**Methods:**

A review of devices for the purification of the air was carried out between January-April 2022. Four different types of air purifiers were considered based on the adopted technologies: I) HEPA filters and UV lamps; II) only with HEPA filters; III) only UV lamps and IV) those using other technologies. For each group, based on the CADR (Clean Air Delivery Rate) provided by the producers, the optimal real surface area of the room to use the device was calculated, referring to the standard EN779:2012. This value was compared with the recommended area of the room declared by the producers. Descriptive statistics and Wilcoxon matched pair test used for comparisons. The significance level was set at p < 0.05.

**Results:**

The analysis was carried out on 252 devices; I) 52 had HEPA filters + UV lamps, with a recommended mean room area of 40 m2 (IQR 49.75), II) 142 devices had only HEPA filters with 52.5 m2 (IQR 46.75), III) 27 devices only UV lamps, 40 m2 (IQR 105), IV) 31 devices with other technologies, 54 m2 (IQR 84.2). As required by EN779:2012, the effective area of activity was calculated using CADR x 0.075: the medians of the 4 groups were I) 12 m2 (IQR 16.5), II) 15.83 m2 (IQR 26.4), III) 4.5 m2 (IQR 22.5), IV) 7.5 m2 (IQR 21.53), respectively. Comparing declared and calculated CADR values, all the groups showed significant differences (p < 0.05).

**Conclusions:**

Results show that recommended surfaces derived from CADR declared by producers largely overestimate the real volume of the room that devices can purify, whatever the technology used.

**Key messages:**

• There’s no correspondence between recommended area of room to be sanitized indicated by producers of air purifiers and area that they are actually able to sanitize, which is significantly lower.

• It is necessary to be aware of the difference between data indicated by producers and real data, in order to purchase a device that actually corresponds to dimensional needs of the environment itself.

